# The Matthew effect in environmental science publication: A bibliometric analysis of chemical substances in journal articles

**DOI:** 10.1186/1476-069X-10-96

**Published:** 2011-11-10

**Authors:** Philippe Grandjean, Mette L Eriksen, Ole Ellegaard, Johan A Wallin

**Affiliations:** 1 Department of Environmental Medicine, and University Library, University of Southern Denmark, Odense, Denmark

## Abstract

**Background:**

While environmental research addresses scientific questions of possible societal relevance, it is unclear to what degree research focuses on environmental chemicals in need of documentation for risk assessment purposes.

**Methods:**

In a bibliometric analysis, we used SciFinder to extract Chemical Abstract Service (CAS) numbers for chemicals addressed by publications in the 78 major environmental science journals during 2000-2009. The Web of Science was used to conduct title searches to determine long-term trends for prominent substances and substances considered in need of research attention.

**Results:**

The 119,636 journal articles found had 760,056 CAS number links during 2000-2009. The top-20 environmental chemicals consisted of metals, (chlorinated) biphenyls, polyaromatic hydrocarbons, benzene, and ethanol and contributed 12% toward the total number of links- Each of the top-20 substances was covered by 2,000-10,000 articles during the decade. The numbers for the 10-year period were similar to the total numbers of pre-2000 articles on the same chemicals. However, substances considered a high priority from a regulatory viewpoint, due to lack of documentation, showed very low publication rates. The persistence in the scientific literature of the top-20 chemicals was only weakly related to their publication in journals with a high impact factor, but some substances achieved high citation rates.

**Conclusions:**

The persistence of some environmental chemicals in the scientific literature may be due to a 'Matthew' principle of maintaining prominence for the very reason of having been well researched. Such bias detracts from the societal needs for documentation on less well known environmental hazards, and it may also impact negatively on the potentials for innovation and discovery in research.

## Background

As thousands of potentially toxic chemicals are being released into the environment, there is a need to document their persistence, dissemination, biomagnification and toxic effects.

In the early 1980s, the US National Research Council completed an extensive study on toxicity testing and found that 78% of the industrial chemicals most commonly produced had not even been minimally tested for toxicity [1]. A follow-up study by the Environmental Defense Fund [2] over ten years later showed little improvement, as did a more detailed study by the U.S. Environmental Protection Agency (EPA) [3]. A voluntary testing program has been initiated in collaboration with the chemical industry to develop minimum toxicity data for 3,000 high-production volume chemicals. However, this effort has been derailed due to delayed, incomplete, and poor-quality data submissions by the chemical producers [4]. Information from the European Chemical Agency also shows that gaps in safety data remain, and that little has been done to mend the problem [5]. Since 2008, the EPA has conducted risk-based prioritizations for several thousand chemicals of potential concern [6], again highlighting the lack of information on environmental dissemination and toxicity available.

Our goal was to examine the literature published in environmental science journals to identify the chemicals that had attracted the most research attention as well as possible trends over time. Although the published literature may not fully represent the research that has actually been conducted [7], it does reflect the information readily available to the public and the academic community about environmental chemicals. The easy availability of scientific literature through the internet facilitates the retrieval of information on environmental chemicals, and it also allows large-scale bibliometric analyses. Several such studies have been carried out in the recent past with a focus on research carried out by the U.S. Environmental Protection Agency [8], in a particular region [9], or published in a particular journal [10]. To obtain an overview of international research on environmental chemicals, we focused on scientific journals that publish articles on environmental science, toxicology, and related fields. We chose to use standard bibliographic databases available on the internet to identify the chemical substances addressed in academic research and published in peer-reviewed journals.

## Methods

From 2000 to 2009, the ISI Web of Science http://apps.isiknowledge.com lists a total of 274 journals within the subject categories "Environmental Science", "Public, Environmental and Occupational Health" and "Toxicology". In the SciFinder data base https://scifinder.cas.org, sixty of the journals were not associated with any Chemical Abstracts Service (CAS) numbers, and less than half of the articles in 89 journals related to at least one CAS number. After excluding these 149 journals, as well as 47 not included in the PubMed Medline database http://www.nlm.nih.gov/bsd/pmresources.html#journals, 78 journals were retained for bibliometric analysis based on their coverage of chemical substances (Additional file 1).

SciFinder was then used to obtain the CAS numbers for the articles published in these journals. The CAS numbers from all articles published in 2000-2009 were downloaded. After sorting the data, we calculated the total number of links for each CAS number extracted from the journals during this 10-year period so we could rank the chemicals according to the number of links, i.e., number of publications that referred to the specific substance. As the CAS numbers represent chemicals in a broad sense, not just industrial chemicals, the chemical names and common names for the most frequently covered CAS numbers were identified, so that elements, isotopes, and biochemical substances could be separated. A list of the environmental chemicals and the most commonly covered 'other' chemicals was generated, allowing us to rank the chemicals based on the degree of attention in the environmental science literature.

Although SciFinder also covers publications before 2000, it only includes citation data from 1996. A different approach was therefore utilized to obtain information on pre-2000 journal articles. In the Web of Science, we searched the number of publications within the three subject categories listed above, with the common name of each chemical of interest listed in the title of the article. The title search approach was considered appropriate, as this would ensure that the chemical selected was the focus of the articles identified. Although this method would likely result in incomplete data on the total coverage of the substances in the scientific literature, publication numbers identified for 2000-2009 and pre-2000 would be appropriate reflections of possible time trends.

To examine the possible association between prominence of environmental chemicals and publication in prestigious journals, the ISI 2009 Five-Year Impact Factor was used to rank the 78 journals (i.e., based on the average number of times published articles over the past five years had been cited in 2009).

If the number of publications on environmental chemicals grow at the same rate, the size distribution will converge toward Gibrat's law and will approach the Pareto distribution [11]. A linear dependence will therefore exist in a log-log plot between number of publications and rank. A deviation from this dependence signals that size matters and hence that a possible Matthew effect may exist in publications on environmental chemicals. Deviation from the Pareto distribution was tested by regression analysis using the data for the top-100 chemicals with inclusion of a second-degree term [11].

## Results

**Figure 1:**
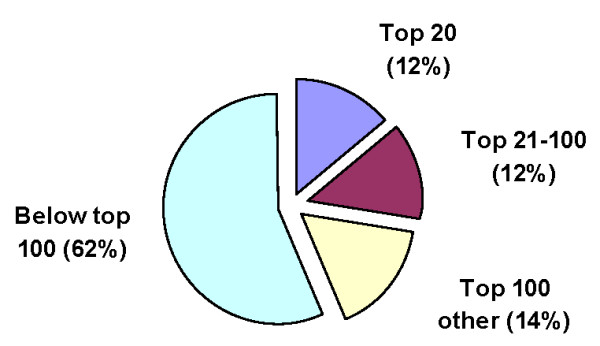
**Relative numbers of publications that refer to the top-20 toxic substances, the next 80, the most common other substances, and the less prominent substances in environmental science journals during 2000-2009**.

The 78 journals selected published a total of 119,636 articles with CAS number links during the ten-year period covered. These articles represented a total of 760,056 CAS references in SciFinder, thus corresponding to an average slightly above six links per article. The 100 most prominent environmental chemicals were each covered in at least 600 articles, i.e., an average of five each month during the ten-year period, with a total of 180,822 SciFinder references, thus representing 24% of the total number of links (Figure 1). One-half of these links referred to the top-20 substances. Although we translated only the first 250 CAS numbers into common names in order to exclude other commonly covered substances, such as hydrogen, glutathione, and P450 enzymes from consideration, the findings suggest that the top-100 substances may represent about one-third of the total number of links. The data base includes links to several thousand CAS numbers, and most substances below the top-100 are each covered in comparatively few articles.

The 20 most commonly studied environmental chemicals are shown in Table 1. Between 2,000 and 10,000 articles addressed each of them during the ten-year period. This corresponds to 200-1,000 articles per year or 4-20 articles every week. Although the numbers of CAS references from different substances cannot be added, as many articles dealt with more than one substance, the total sum of article links (91,844) for the top-20 chemicals is sizeable and corresponds to 12% of all CAS number links. The chemicals ranked 21-100 are listed in Additional file 2. Substances with a rank just below 100 had about 600 links, and each time one of these substances was covered in a publication, lead and copper would have appeared in at least 15 articles. The top-100 substances include numerous related congeners, isomers and otherwise similar chemicals which, to a large extent were probably covered by the same articles. If these possible duplicates are taken into account, perhaps less than 60 compounds, or groups of compounds, constitute the most commonly studied chemicals, with more than 600 article links during the 10-year period.

**Table 1:** Top-20 chemicals covered by environmental journals during 2000-2009.

Rank	Substance name	Number of links 2000-2009	Fraction of total titles published > 2000	Average number of citations*
1	Copper	9573	53	11.5
2	Lead	8926	42	9.9
3	Zinc	8323	45	11.9
4	Cadmium	8199	41	11.7
5	Iron	6948	60	13.6
6	Nickel	5395	49	13.0
7	Chromium	5123	54	12.3
8	Arsenic	4626	74	14.4
9	Mercury	4399	51	11.8
10	Manganese	4311	53	10.8
11	1,1'-Biphenyl	3897	42	14.5
12	Aluminum	3416	31	6.5
13	Benzo[a]pyrene	2842	66	9.1
14	Phenanthrene	2669	72	12.4
15	Pyrene	2447	43	9.8
16	Naphthalene	2206	53	13.4
17	Ethanol	2197	42	8.8
18	Cobalt	2143	49	10.1
19	Benzene	2113	47	10.1
20	Fluoranthene	2091	54	8.3

		Sum = 91,844	Average = 51	Average = 11.2

Number of article links from SciFinder for 2000-2009, the relative number of publications during this decade compared to the total number since 1900, and the average number of citations per article for the given substance during the period 2000-2009 from the Web of Science.

**Figure 2:**
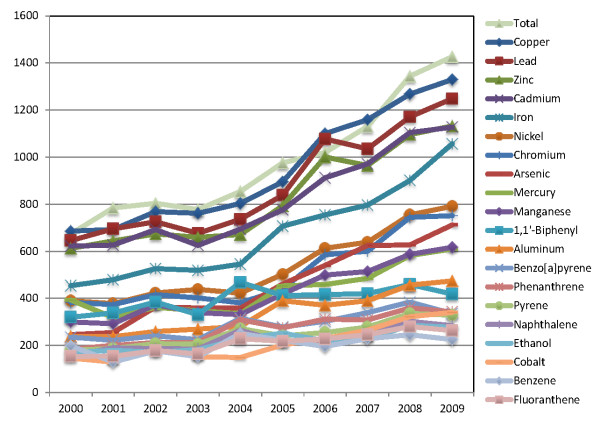
**Total numbers of publications on the top-20 toxic substances in environmental science journals during 2000-2009, as compared to the total average number for each journal**.

During the first ten years of the millennium, the annual number of articles concerning the top 20 substances increased by 70%, but the relative abundance decreased slightly due to a doubling in the total numbers of links (Figure 2). The relative emphasis on each of these chemicals changed minimally, with the ranking of the top-20 substances in 2000 being similar to the one in 2009 (p = 0.85 by Wilcoxon test, Additional File 3).

To compare this with the pre-2000 coverage of the same chemicals, we extracted from the Web of Science the number of publications with the top 20 substances in the title field and those that were published in journals belonging to the same topic groupings, accrued during 1900-1999. On average, about half of the publications available on these substances was published after the millennium (Table 1). There were some variations, with arsenic becoming more prominent, and aluminum less, after 2000. Also, polyaromatic hydrocarbons tended to appear more often in the recent article titles. Overall, the chemicals most commonly studied from 2000 to 2009 had already attracted much research during the previous century. For comparison, when 'environment*' was used as a search item under publication name in a general search, we found 76,701 publications before 2000 and 68,418 in 2000-2009, i.e., 47% published after 2000. These data therefore reflect a continuously high or even increasing rate of publication in this field.

To examine the publication frequencies for chemicals that differ substantially in regard to available information, we first focused on substances that could be considered having been thoroughly studied. Thus, the European Environment Agency in 2002 included six chemicals in its series of case studies on so-called late lessons of early warnings [12]. These chemical hazards were extensively represented in scientific studies before 2000. On the other extreme, environmental toxicants that have been overlooked were identified in 2006 by the U.S. EPA in a listing of high-production chemicals in particular need of scientific documentation [13]. Thirteen compounds were listed as high priority, in regard to both hazard data and exposure information.

The first group of six well studied substances attracted a total of 8,267 links during 2000-2009, or an average of at least 10 articles per substance every month (Table 2). In contrast, when using the CAS numbers listed by the EPA, the secondary tier of high-priority substances had a total of only 352 links to articles published in the same 78 journals from 2000-2009, or an average of 3 per month for the entire group (Table 3). Five of these thirteen high-priority substances were not encountered at all in the journals during the period searched. As we cannot confirm or deny that some articles may have been recorded under other CAS numbers, the numbers may be underestimated, though this problem may relate to many other chemicals as well. The annual number of CAS links from 2007-2009, i.e., from articles published after the EPA listing, was 39, as compared to 36 during 2000-2006. A total of 34 links for all of these substances during 2010 also did not support any overall increasing trend. However, the links to triclocarban increased from a total of eight during 2000-2006 to 35 during 2007-2010. Still, the already well-studied substances continued to motivate many more publications than did the substances deemed to constitute a high priority for research.

**Table 2:** Total numbers of articles on 'early warnings' substances in the title in environmental and toxicology journals during 2000-2009.

Name*	Number of links
Polychlorinated biphenyls	3,897
Benzene	2,113
Sulfur dioxide	1,161
Tributyl tin	308
Methyl-tert-butyl ether	497
Diethylstilbestrol	291

	Sum = 8,267

Substance names from the European Environment Agency, number of articles from the Web of Science.

**Table 3:** Total numbers of articles on high-priority substances in environmental and toxicology journals during 2000-2009.

Name	CAS number	Number of links
1,3-Dichlorobenzene	541-73-1	242
Methane, bromochloro-	74-97-5	45
Triclocarban	101-20-2	21
Hexabromocyclododecane	3194-55-6	11
Monoglyme	110-71-4	10
Diglyme	111-96-6	10
1H-1,2,4-Triazole	288-88-0	9
Ethanol, 2-(2-aminoethoxy)-	929-06-6	3
2H-3,1-Benzoxazine-2,4(1H)-dione	118-48-9	1
Quaternary ammonium compounds, benzyl (hydrogenated tallow alkyl) dimethyl, chlorides	61789-72-8	0
Quaternary ammonium compounds, benzylbis (hydrogenated tallow alkyl) methyl, chlorides	61789-73-9	0
Quaternary ammonium compounds, benzyl-C12-16-alkyldimethyl, chlorides	68424-85-1	0
Phosphonic acid, dibutyl ester	1809-19-4	0

		Sum = 352

These chemicals were considered to be of highest priority for research by the U.S. Environmental Protection Agency, and CAS numbers were used to identify the number of articles in SciFinder.

To explore whether journal impact factor played a role, we separated the journals into tertile groups according to their Five-Year Impact Factor. Although ethanol was relatively more frequently represented in low-impact journals, the opposite was true for (chlorinated) biphenyls, arsenic and mercury. However, no overall tendency in the rankings of the overall top-20 chemicals was found when comparing low impact factor (< 1.88) and high (> 2.92) (p = 0.15, Wilcoxon, Supplementary table 3). The journals in the top tertile of impact factors contributed 52,306 (or 58%) to the CAS references of the top-20 chemicals, a proportion only slightly higher than their total share of CAS number links (56%) for these journals.

**Figure 3:**
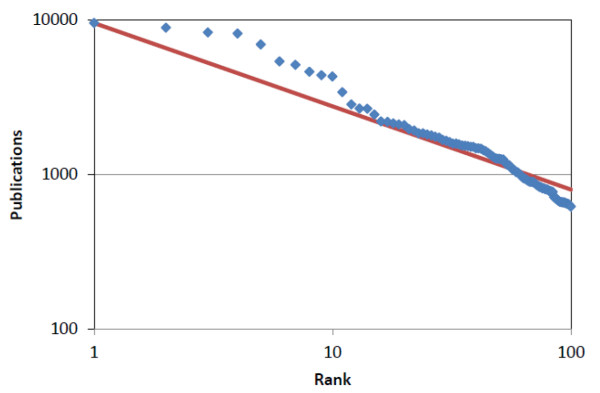
**Log-log plot of number of CAS links and rank for the 100 most prominent environmental chemicals**. The straight line represents the Pareto distribution.

Figure 3 shows a log-log plot for rank and number of CAS links based on the data from Table 1 and Supplementary Table 2. The regression line reflects Gibrat's law, which would predict that numbers of links increase at the same rate. Most of the chemicals with a rank less than 10 show a disproportionately higher number of publications, while chemicals with a rank approaching 100 had fewer CAS links than would be consistent with proportional growth. The deviation from the linear relationship was examined in a regression analysis, where a second-degree term has a p value less than 0.001, thus supporting that chemicals with a low rank attract much larger numbers of publications, as compared with less prominent chemicals.

## Discussion

The results obtained in this study suggest a serious bias in publications on environmental chemicals. While regulatory agencies request information on poorly studied, potentially serious environmental hazards, the publications in scientific journals emphasize a relatively small, selective number of highly prominent chemicals, about which large numbers of articles were published already during the past century. The focus on inorganic substances is noteworthy; the top-10 substances are all metals or metalloids. Copper is a well-established ecological hazard, and lead toxicity has been known since antiquity. As also indicated by the Web of Science search, all the most popular chemicals had substantial publication numbers also in the past century. All told, tens of thousands of articles have been published in scientific journals on these substances.

While the well-known chemicals remained a focal point in published research reports, the substances from the EPA priority list attracted only a small number of publications, if any. The long-term prominence of substances commonly covered in articles in environmental science journals therefore ignores the needs of regulatory agencies like the U.S.EPA. The enormous contrast in publication coverage suggests that regulatory needs for documentation are not impacting environmental science priorities.

The retained focus on a limited number of prominent chemicals may be due to continued environmental or occupational exposures, in spite of any preventive efforts [12]. Although that may be true for many of the commonly studied chemicals, especially those that are persistent in the environment and in the human body, the question is whether incomplete or lack of intervention should justify research focus on issues that have already been thoroughly covered by extensive research. A key question is whether the desire for a solid proof on certain chemicals should overrule the need for basic information on other potential hazards. Still, some well-studied substances may represent an important scientific paradigm or useful reference, thereby justifying their inclusion in some research studies. We did not attempt to evaluate the justification of the focus in individual studies, nor did we attempt to assess the validity or impact of individual articles.

This study relies on bibliographic databases being routinely available to obtain information on the coverage of chemicals by scientific journals in the environmental science field. Because of the reliability of the data bases used, the results are likely to represent overall tendencies of scientific attention to different chemical compounds. However, each CAS number link may refer to an article with only limited measurement data or, on the other extreme, a comprehensive review. The depth of our study is based solely on the mere number of articles located. While SciFinder is unlikely to underestimate the numbers of publications, unless a substance is commonly related to more than one CAS number, e.g., due to isomers, impurities, or mixtures, the numbers obtained from our Web of Science title searches are most likely underestimates, as all relevant articles may not have included the name of the substance in the title of the publication. Assuming that this error would be unlikely to change much over time, the comparisons of relative numbers before and after 2000 would still be representative.

A previous study on European environmental research listed as many as 711 journals [9] publishing in the field of environmental research. However, most of these journals must have published only a small number of the 6329 references identified, and only a minority would have dealt with environmental chemicals. Also, some general science or medicine journals occasionally publish articles on environmental chemicals, and these publications were not included in the searches based only on the major 78 environmental and toxicology journals. Thus, while the total number of articles covered by the journals included in the present study was probably underestimated, it is unlikely that any serious bias would have occurred by our selection of topic-specific journals.

A certain amount of inertia in regard to the choice of research topics is undoubtedly present at universities, among researchers, and funding agencies [14]. Tradition and availability of established methods, existing instrumentation, and experience no doubt play a role. The metals, for example, can be easily and inexpensively measured by a common instrument called an atomic absorption spectrometer. Modern equipment, such as inductively-coupled plasma mass spectrometry, can even measure several metals at the same time. Likewise, some of the tar chemicals can be measured by gas chromatography, another inexpensive instrument that is widely available in research laboratories worldwide. Hundreds of analyses can be produced in a week, perhaps enough to justify a manuscript for a scientific journal. When inexpensive routine methods are available, who can blame researchers for taking advantage of generating another research report?

If feasibility is a criterion, then the choice is obvious. Thesis advisors, who have themselves studied metals, and who have established methods for their analysis, will likely recommend metals research to their students, rather than a more cumbersome project on lesser known environmental chemicals, where the outcome may be difficult to predict.

Within scientific communities focusing on heavy metals, aromatic hydrocarbons, or chlorinated organic compounds, citations are likely to be high, to the extent that members of these communities are highly active, participate in specialized conferences, and publish frequently. They are likely to cite their own work and the publications by close colleagues that support their research perspective. Some support for this notion is apparent from Table 1, where arsenic and biphenyls have twice the number citations of aluminum. Coverage in high-impact journals with a wide readership potentially will lead to these articles being more likely to be cited [15,16], as opposed to those of less prominence, thereby perpetuating the assumed importance of the research. Again, arsenic and biphenyls were more frequently covered in the upper tertile of environmental science journals, but this tendency was not apparent for other environmental chemicals.

Merton [16] described the self-prophetic bias that maintains a continued prominence of a small number of scientists and their publications. He dubbed this a 'Matthew' effect, referring to the New Testament ('For unto every one that hath shall be given, and he shall have abundance: but from him that hath not shall be taken away even that which he hath'). An extension of this principle assigns importance to widely published topics, in our case specific environmental chemicals, for the very reason that they are already widely covered in the literature. Whether or not they are persistent in the environment or the human body, they become persistent in the scientific literature. The tens of thousands of articles on copper, lead, cadmium, and other prominent environmental hazards testify to the enormous investments in studying, reporting, and publishing on these popular substances. In other words, fame reinforces itself [11].

Such self-affirming, or perhaps self-serving, bias can impede the progress of research in new fields and studies covering new substances, thereby resulting only in incremental advances, at best, from the research investments. The EPA priority chemicals continue to be ignored, and scientific discovery is impeded by a narrow focus on a limited number of favored chemicals. As noted by Merton [16], a disproportionate focus on prominent substances in environmental science journals may be nurtured by the self-prophecy associated with an increased number of citations in the scientific literature.

Several factors likely play a role in addition to the motivation of researchers to tackle challenging environmental health problems. Apart from mere inertia within the research institutions, a major determinant is the accessibility of federal, private or other funding for research in the less apparent and only suspected hazards. Some degree of inertia probably also exists within funding agencies. Research on major environmental chemicals is easier to evaluate due to the availability of experts. They may tend to recommend that further research on their favorite substances is warranted, one possible advantage being that additional studies will generate more attention and perhaps citations of their own publications. For the agencies, an attractive aspect is that the research outcome is easier to predict and to interpret as well as probably being less expensive. If focusing on poorly-known substances, the risk is greater that the detection method is problematic, the doses used are inappropriate, or that some other, unexpected problem will render the results invalid or uninformative. However, for these very reasons, research on less-commonly studied chemicals is highly needed, not the least to pave the way for more definite studies and to facilitate innovation and unexpected discoveries.

## Conclusions

This bibliometric study indicates that a small number of environmental chemicals may be persistent, not only in the environment or in the human body, but also in the scientific literature. Furthermore, the results show that the inertia in scientific publication is resilient. We suggest that this tendency relates to a 'Matthew' principle of maintaining prominence for the very reason that these substances have been well researched in the past. Such continued, narrow focus may represent a self-serving bias in science, which thrives upon high citation rates and attention within specialized scientific groups. However, it detracts from the societal needs for documentation on less well known environmental hazards, and it may also adversely impact upon the needs for innovation and discovery in research. Funding agencies, traditions of scientific institutions, and publication practices probably all contribute toward this scientific inertia.

## Abbreviations

CAS: Chemical Abstract Service; EPA: Environmental Protection Agency.

## Competing interests

The authors declare that they have no competing interests.

## Authors' contributions

PG and MLE developed the first study outline, which was extended in collaboration with OE and JAW. MLE conducted the web searches and managed the data base. The results were discussed and interpreted by all authors. PG drafted the first version of the manuscript, to which all authors contributed. All authors approved the final version.

## Supplementary Material

Additional file 1 **Journals covered during 2000-2009**. Journals listed by PubMed and within Web of Science subject categories "Environmental Science", "Public, Environmental and Occupational Health" and "Toxicology", where at least 50% of articles published during 2000-2009 had at least one Chemical Abstracts Service (CAS) number link in SciFinder. The journals were separated into tertile groups based on their 2009 impact factor. Click here for file

Additional file 2 **Most commonly covered chemicals below top-20**. Top 21-100 environmental chemicals with their number of links. Click here for file

Additional file 3 **Details on top-20 chemical ranks**. Rank of top-20 chemicals in high and low impact-factor journals and in 2000 and 2009 Click here for file
