# Supplementary material for: The Matthew effect in environmental science publication: A bibliometric analysis of chemical substances in journal articles
Source: Environ Health. 2011 Nov 10;10:96. doi: 10.1186/1476-069X-10-96 (PMC3229577; doi:10.1186/1476-069X-10-96)
Supplement: Additional file 1 — Journals covered during 2000-2009. Journals listed by PubMed and within Web of Science subject categories "Environmental Science", "Public, Environmental and Occupational Health" and "Toxicology", where at least 50% of articles published during 2000-2009 had at least one Chemical Abstracts Service (CAS) number link in SciFinder. The journals were separated into tertile groups based on their 2009 impact factor. [file 1476-069X-10-96-S1.DOC]

Table 1. Journals listed by PubMed and within Web of Science subject categories “Environmental Science”, “Public, Environmental and Occupational Health” and “Toxicology”, where at least 50% of articles published during 2000-2009 had at least one Chemical Abstracts Service (CAS) number link in SciFinder. The journals were separated into tertile groups based on their 2009 impact factor.

| **Journal Name** | **Total number of articles** | **Articles with CAS numbers** | | **5-Year Impact Factor** |
| --- | --- | --- | --- | --- |
|  |  | **Number** | **Percent** |  |
| Environmental Health Perspectives | 4,411 | 2,374 | 53.82 | 7.103 |
| Critical Review of Toxicology | 272 | 136 | 50.00 | 6.481 |
| Environmental Science & Technology | 13,443 | 9,740 | 72.45 | 5.438 |
| Toxicological Sciences | 2,998 | 2,569 | 85.69 | 4.845 |
| Journal of Toxicology and Environmental Health-Part B | 180 | 93 | 51.67 | 4.841 |
| Water Research | 5,335 | 4,145 | 77.69 | 4.828 |
| Journal of Hazardous Materials | 7,240 | 5,962 | 82.35 | 4.360 |
| Toxicology and Applied Pharmacology | 2,875 | 2,431 | 84.56 | 4.042 |
| Drugs | 1,669 | 1,312 | 78.61 | 4.006 |
| Environmental Pollution | 4,159 | 3,494 | 84.01 | 3.904 |
| Aquatic Toxicology | 1,635 | 1,492 | 91.25 | 3.835 |
| Chemosphere | 8,233 | 7,487 | 90.94 | 3.762 |
| Chemical Research in Toxicology | 2,211 | 2,023 | 91.50 | 3.555 |
| Drug Safety | 561 | 417 | 74.33 | 3.552 |
| Mutagenesis | 730 | 487 | 66.71 | 3.530 |
| Toxicology Letters | 2,577 | 1,919 | 74.47 | 3.400 |
| Science of the Total Environment | 5,461 | 3,529 | 64.62 | 3.399 |
| Environmental Research | 1,318 | 929 | 70.49 | 3.326 |
| Toxicology | 3,236 | 2,361 | 72.96 | 3.297 |
| Journal of Environmental Science and Health Part C | 90 | 53 | 58.89 | 3.268 |
| Neurotoxicology | 1,129 | 959 | 84.94 | 3.126 |
| Reviews of Environmental Contamination and Toxicology | 161 | 92 | 57.14 | 3.025 |
| Chemico-Biological Interactions | 1,835 | 1,568 | 85.45 | 3.012 |
| Journal of Environmental Quality | 3,060 | 1,969 | 64.35 | 2.981 |
| Reproductive Toxicology | 1187 | 775 | 65.29 | 2.963 |
| Waste Management | 1,947 | 1,132 | 58.14 | 2.924 |
| Ecotoxicology | 645 | 484 | 75.04 | 2.922 |
| Neurotoxicology and Teratology | 867 | 541 | 62.40 | 2.920 |
| Inhalation Toxicology | 1,209 | 757 | 62.61 | 2.882 |
| Mutation Research-Genetic Toxicology and Environmental Mutagenesis | 1,823 | 1,346 | 73.83 | 2.740 |
| Environmental Toxicology and Chemistry | 3,703 | 3,240 | 87.50 | 2.734 |
| Ecotoxicology and Environmental Safety | 1,710 | 1,426 | 83.39 | 2.674 |
| Environmental and Molecular Mutagenesis | 851 | 554 | 65.10 | 2.649 |
| Archives of Toxicology | 1,085 | 943 | 86.91 | 2.626 |
| Alcohol | 9,807 | 4,967 | 50.65 | 2.532 |
| International Journal of Hygiene and Environmental Health | 688 | 348 | 50.58 | 2.526 |
| Toxicologic Pathology | 225 | 196 | 87.11 | 2.475 |
| Therapeutic Drug Monitoring | 1,220 | 1,092 | 89.51 | 2.472 |
| Toxicology In Vitro | 1,488 | 1,288 | 86.56 | 2.461 |
| Environmental Science and Pollution Research | 722 | 448 | 62.05 | 2.448 |
| Toxicon | 2,733 | 1,800 | 65.86 | 2.437 |
| Xenobiotica | 864 | 784 | 90.74 | 2.429 |
| Journal of Contaminant Hydrology | 1,122 | 736 | 65.60 | 2.345 |
| Journal of Environmental Monitoring | 1,989 | 1,256 | 63.15 | 2.236 |
| Biomarkers | 487 | 355 | 72.90 | 2.217 |
| Regulatory Toxicology and Pharmacology | 930 | 595 | 63.98 | 2.093 |
| Marine Environmental Research | 1,092 | 722 | 66.12 | 2.060 |
| Environmental Toxicology | 761 | 642 | 84.36 | 2.041 |
| Journal of Applied Toxicology | 825 | 717 | 86.91 | 1.927 |
| Journal of The Air & Waste Management Association | 1,496 | 1,064 | 71.12 | 1.924 |
| Journal of Toxicology and Environmental Health-Part A | 1,633 | 1,198 | 73.36 | 1.897 |
| Environmental Geochemistry and Health | 510 | 426 | 83.53 | 1.885 |
| SAR and QSAR an Environmental Research | 438 | 243 | 55.48 | 1.869 |
| The International Journal of Phytoremediation | 304 | 275 | 90.46 | 1.852 |
| Journal of Biochemical and Molecular Toxicology | 476 | 414 | 86.97 | 1.847 |
| Cell Biology and Toxicology | 396 | 342 | 86.36 | 1.839 |
| Human & Experimental Toxicology | 1,013 | 716 | 70.68 | 1.667 |
| Radiation and Environmental Biophysics | 435 | 235 | 54.02 | 1.644 |
| Journal of Analytical Toxicology | 1,543 | 1,051 | 68.11 | 1.569 |
| Experimental and Toxicologic Pathology | 611 | 451 | 73.81 | 1.542 |
| Journal of Environmental Radioactivity | 1,728 | 1,198 | 69.33 | 1.485 |
| Environmental Monitoring and Assessment | 2,828 | 1,805 | 63.83 | 1.387 |
| Drug and Chemical Toxicology | 382 | 320 | 83.77 | 1.324 |
| Journal of Environmental Science and Health Part A | 1,932 | 1,574 | 81.47 | 1.301 |
| International Journal of Toxicology | 589 | 455 | 77.25 | 1.281 |
| Water Science and Technology | 7,305 | 4,625 | 63.31 | 1.228 |
| Toxicology and Industrial Health | 408 | 333 | 81.62 | 1.090 |
| Journal of Environmental Science And Health Part B | 828 | 742 | 89.61 | 1.064 |
| Journal of Environmental Sciences (China) | 1,800 | 1,463 | 81.28 | 1.012 |
| Immunopharmacology and Immunotoxicology | 605 | 420 | 69.42 | 0.941 |
| Water Environment Research | 1,416 | 759 | 53.60 | 0.935 |
| Isotopes in Environmental and Health Studies | 362 | 232 | 64.09 | 0.924 |
| Biomedical and Environmental Sciences | 615 | 389 | 63.25 | 0.859 |
| Bulletin of Environmental Contamination and Toxicology | 2,923 | 1,868 | 63.91 | 0.841 |
| Environmental Technology | 1,522 | 1,240 | 81.47 | 0.782 |
| Radiation Protection Dosimetry | 3,687 | 1,885 | 51.13 | 0.754 |
| Journal of Environmental Protection and Ecology | 804 | 647 | 80.47 | - |
| Journal of Materials Science | 9,860 | 8,581 | 87.03 | - |
| Total | 163,248 | 119,636 |  |  |
