# Supplementary material for: The Matthew effect in environmental science publication: A bibliometric analysis of chemical substances in journal articles
Source: Environ Health. 2011 Nov 10;10:96. doi: 10.1186/1476-069X-10-96 (PMC3229577; doi:10.1186/1476-069X-10-96)
Supplement: Additional file 2 — Most commonly covered chemicals below top-20. Top 21-100 environmental chemicals with their number of links. [file 1476-069X-10-96-S2.DOC]

Table 2. Top 21-100 environmental chemicals with their number of links

|  | Chemical name | CAS no. | Number  of links |
| --- | --- | --- | --- |
| 21 | Benzene, 1,1'(2,2,2-trichloroethylidene)bis[4-chloro-phenyl] (p,p‘-DDT) | 50-29-3 | 1968 |
| 22 | Anthracene | 120-12-7 | 1931 |
| 23 | Silicon(iv) oxide | 7631-86-9 | 1853 |
| 24 | Chrysene | 218-01-9 | 1850 |
| 25 | Benz[a]anthracene | 56-55-3 | 1818 |
| 26 | 2,3,7,8-Tetrachlorodibenzo-p-dioxin | 1746-01-6 | 1795 |
| 27 | Benzene,1,1'-(2,2-dichloroethenylidene)bis[4-chloro-phenyl] (p,p'-DDE) | 72-55-9 | 1758 |
| 28 | Dibenzo-p-dioxin | 262-12-4 | 1736 |
| 29 | Dibenzofuran | 132-64-9 | 1675 |
| 30 | 1,1'-Biphenyl, 2,2',4,4',5,5'-hexachloro- | 35065-27-1 | 1657 |
| 31 | Selenium atom | 7782-49-2 | 1626 |
| 32 | gamma-Hexachlorocyclohexane (Lindane) | 58-89-9 | 1590 |
| 33 | Fluorene | 86-73-7 | 1586 |
| 34 | Toluene | 108-88-3 | 1568 |
| 35 | Benzo[k]fluoranthene | 207-08-9 | 1543 |
| 36 | Benz[e]acephenanthrylene | 205-99-2 | 1537 |
| 37 | Benzo[ghi]perylene | 191-24-2 | 1528 |
| 38 | Phenol | 108-95-2 | 1513 |
| 39 | Aluminum oxide | 1344-28-1 | 1511 |
| 40 | 1,1'-Biphenyl, 2,3',4,4',5-pentachloro- | 31508-00-6 | 1478 |
| 41 | Carbon monoxide | 630-08-0 | 1469 |
| 42 | 1,1'-Biphenyl, 2,2',3,4,4',5,5'-heptachloro- | 35065-29-3 | 1436 |
| 43 | o-Phenylenepyrene | 193-39-5 | 1413 |
| 44 | Dibenz[a,h]anthracene | 53-70-3 | 1381 |
| 45 | Benzene, hexachloro- | 118-74-1 | 1343 |
| 46 | Trichloroethylene | 79-01-6 | 1309 |
| 47 | 1,1'-Biphenyl, 2,2',3,4,4',5'-hexachloro- | 35065-28-2 | 1280 |
| 48 | Acenaphthene | 83-32-9 | 1267 |
| 49 | Nitric oxide | 10102-43-9 | 1265 |
| 50 | Vanadium | 7440-62-2 | 1257 |
| 51 | 2,2',4,5,5' Pentachlorobiphenyl | 37680-73-2 | 1252 |
| 52 | Atrazine | 1912-24-9 | 1199 |
| 53 | Sulfur dioxide | 7446-09-5 | 1161 |
| 54 | 2,2',5,5'-Tetrachlorobiphenyl | 35693-99-3 | 1154 |
| 55 | Acenaphthylene | 208-96-8 | 1121 |
| 56 | Ethane, 1,1-dichloro-2,2-bis(4-chlorophenyl)- (p,p’-DDD) | 72-54-8 | 1078 |
| 57 | Chlorine | 7782-50-5 | 1066 |
| 58 | 1,1'-Biphenyl, 2,3,3',4,4'-pentachloro- | 32598-14-4 | 1036 |
| 59 | 1,1'-Biphenyl, 2,4,4'-trichloro- | 7012-37-5 | 1035 |
| 60 | alpha-Hexachlorocyclohexane (Lindane) | 319-84-6 | 1007 |
| 61 | o,o-Diethyl-o-(3,5,6-trichloro-2-pyridyl)phosphorothioate (Clorpyrifos) | 2921-88-2 | 977 |
| 62 | Phenol, 4,4'-(1-methylethylidene)bis- (Bisphenol A) | 80-05-7 | 952 |
| 63 | Octachlorodibenzo-p-dioxin | 3268-87-9 | 941 |
| 64 | Molybdenum | 7439-98-7 | 930 |
| 65 | 1,2,3,4,10,10-Hexachloro-6,7-epoxy-1,4,4a,5,6,7,8,8a-octahydro-1,4-endo-exo-5,8-dimethanonaphthalene (Dieldrin) | 60-57-1 | 915 |
| 66 | Nitrogen dioxide | 10102-44-0 | 900 |
| 67 | Tetrachloroethylene | 127-18-4 | 898 |
| 68 | Chloroform | 67-66-3 | 897 |
| 69 | Formaldehyde | 50-00-0 | 885 |
| 70 | Cyclohexane, 1,2,3,4,5,6-hexachloro- | 319-85-7 | 874 |
| 71 | Methyl alcohol | 67-56-1 | 856 |
| 72 | Nitrogen oxide | 11104-93-1 | 844 |
| 73 | 1,1'-Biphenyl, 2,3,3',4,4',5-hexachloro- | 38380-08-4 | 832 |
| 74 | Antimony | 7440-36-0 | 830 |
| 75 | 2,3,4,7,8-Pentachlorodibenzofuran | 57117-31-4 | 816 |
| 76 | 2,3,7,8-Tetrachlorodibenzofuran | 51207-31-9 | 814 |
| 77 | 1,1'-Biphenyl,3,3',4,4',5-pentachloro- | 57465-28-8 | 804 |
| 78 | 1,1'-Biphenyl, 3,3',4,4'-tetrachloro- | 32598-13-3 | 802 |
| 79 | 1,2,3,4,6,7,8-Heptachlorodibenzodioxin | 35822-46-9 | 800 |
| 80 | 2-Propanone | 67-64-1 | 784 |
| 81 | Dibenzofuran, octachloro- | 39001-02-0 | 781 |
| 82 | Benzene, ethyl- | 100-41-4 | 778 |
| 83 | Phenol, pentachloro- | 87-86-5 | 772 |
| 84 | 1,2,3,7,8-Pentachlorodibenzodioxin | 40321-76-4 | 715 |
| 85 | 1,1'-Biphenyl, 2,2',3,3',4,4',5-heptachloro- | 35065-30-6 | 702 |
| 86 | Hydrogen chloride | 7647-01-0 | 701 |
| 87 | 2,2',4,4'-tetrabromodiphenyl ether | 5436-43-1 | 682 |
| 88 | 1,2,3,4,7,8-Hexachlorodibenzodioxin | 39227-28-6 | 674 |
| 89 | 1,2,3,6,7,8-Hexachlorodibenzo-p-dioxin | 57653-85-7 | 666 |
| 90 | 1,2,3,4,6,7,8-Heptachlorodibenzofuran | 67562-39-4 | 663 |
| 91 | 1,2,3,4,7,8-Hexachlorodibenzofuran | 70648-26-9 | 661 |
| 92 | 1,1,1-Trichloro-2-(2-chlorophenyl)-2-(4-chlorophenyl)ethane (o,p’-DDT) | 789-02-6 | 660 |
| 93 | 1,2,3,7,8-Pentachlorodibenzofuran | 57117-41-6 | 659 |
| 94 | 1,2,3,7,8,9-Hexachlorodibenzo-p-dioxin | 19408-74-3 | 653 |
| 95 | Sodium hydroxide | 1310-73-2 | 650 |
| 96 | Bis(2-ethylhexyl)phthalate | 117-81-7 | 647 |
| 97 | 1,2,3,6,7,8-Hexachlorodibenzofuran | 57117-44-9 | 642 |
| 98 | Benzene, 1,2-dimethyl- | 95-47-6 | 631 |
| 99 | 2,3,4,6,7,8-Hexachlorodibenzofuran | 60851-34-5 | 621 |
| 100 | 1,2,3,4,7,8,9-Heptachloro-dibenzofuran | 55673-89-7 | 619 |
