# Supplementary material for: The Matthew effect in environmental science publication: A bibliometric analysis of chemical substances in journal articles
Source: Environ Health. 2011 Nov 10;10:96. doi: 10.1186/1476-069X-10-96 (PMC3229577; doi:10.1186/1476-069X-10-96)
Supplement: Additional file 3 — Details on top-20 chemical ranks. Rank of top-20 chemicals in high and low impact-factor journals and in 2000 and 2009 [file 1476-069X-10-96-S3.DOC]

Table 3. Rank of top-20 chemicals in high and low impact-factor journals and in 2000 and 2009.

| Overall rank | Substance name | Number of articles | Rank in high IF journals | Rank in low IF journals | Rank in 2000 | Rank in 2009 |
| --- | --- | --- | --- | --- | --- | --- |
| 1 | Copper | 9573 | 2 | 1 | 1 | 1 |
| 2 | Lead | 8926 | 1 | 2 | 2 | 2 |
| 3 | Zinc | 8323 | 3 | 3 | 4 | 3 |
| 4 | Cadmium | 8199 | 4 | 4 | 3 | 4 |
| 5 | Iron | 6948 | 5 | 5 | 5 | 5 |
| 6 | Nickel | 5395 | 7 | 6 | 7 | 6 |
| 7 | Chromium | 5123 | 8 | 7 | 8 | 7 |
| 8 | Arsenic | 4626 | 6 | 13 | 12 | 9 |
| 9 | Mercury | 4399 | 10 | 15 | 6 | 11 |
| 10 | Manganese | 4311 | 12 | 9 | 11 | 10 |
| 11 | 1,1'-Biphenyl | 3897 | 11 | 33 | 9 | 14 |
| 12 | Aluminum | 3416 | 13 | 10 | 13 | 12 |
| 13 | Benzo[a]pyrene | 2842 | 16 | 24 | 14 | 18 |
| 14 | Phenanthrene | 2669 | 15 | 19 | 17 | 16 |
| 15 | Pyrene | 2447 | 18 | 23 | 18 | 19 |
| 16 | Naphthalene | 2206 | 21 | 28 | 23 | 21 |
| 17 | Ethanol | 2197 | 63 | 18 | 19 | 22 |
| 18 | Cobalt | 2143 | 22 | 17 | 26 | 17 |
| 19 | Benzene | 2113 | 23 | 26 | 16 | 30 |
| 20 | Fluoranthene | 2091 | 25 | 32 | 24 | 23 |
